# Supplementary material for: Rapid Proteome‐Wide Discovery of Protein–Protein Interactions With ppIRIS
Source: Adv Sci (Weinh). 2026 Mar 12;13(26):e21310. doi: 10.1002/advs.202521310 (PMC13159128; doi:10.1002/advs.202521310)
Supplement: Supplementary file 1 — Supporting File: advs74658‐sup‐0001‐SuppMat.pdf. [file ADVS-13-e21310-s001.pdf]

Supplementary Material for Rapid Proteome-wide Discovery of Protein-Protein Interactions with ppIRIS

Luiz Felipe Piochi[1], Di Tang[2], Johan Malmström[2,3], Yasaman Karami[1], Hamed Khakzad[1,*]

[1] Université de Lorraine, CNRS, Inria, LORIA, F-54000 Nancy, France
[2] Division of Infection Medicine, Department of Clinical Sciences Lund, Faculty of Medicine, Lund University, Lund, Sweden
[3] SciLifeLab, Integrated Structural Biology platform, Structural Proteomics Unit Sweden, Lund University, Lund, Sweden
[*] corresponding author: hamed.khakzad@inria.fr

Supplementary Figures

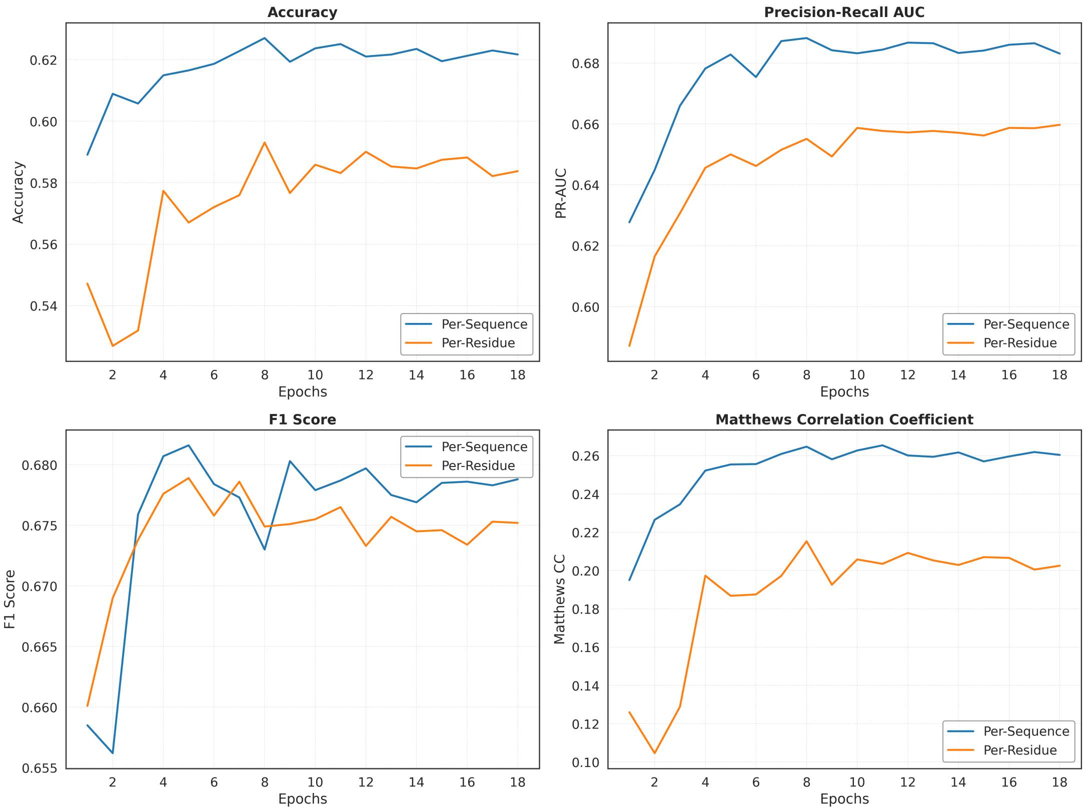

Figure S1: Comparison of performance metrics when using per-sequence versus per-token embeddings on the human gold-standard PPI dataset. While per-token embeddings theoretically capture positional information, our results show they provide minimal additional benefit for PPI prediction while substantially increasing computational demands. We therefore used per-sequence embeddings throughout our study for efficiency.

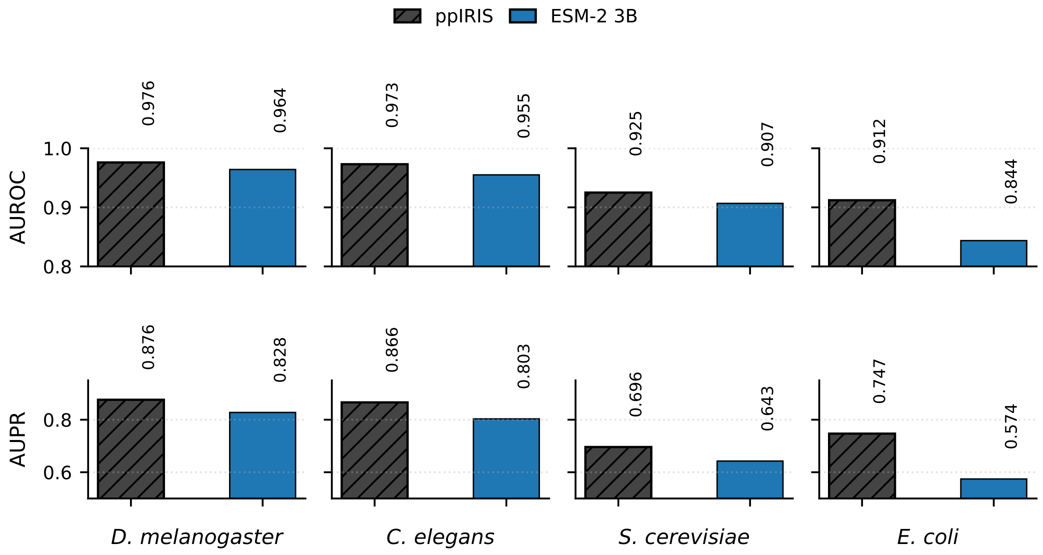

Figure S2: Performance comparison of ppIRIS and ESM-2 3B on D-SCRIPT datasets. Bar charts display the performance of ppIRIS (black hatched bars) versus the ESM3B baseline (blue bars) on the D-SCRIPT test sets for D. melanogaster, C. elegans, S. cerevisiae, and E. coli. The models are evaluated across AUROC (top row) and AUPR (bottom row). ppIRIS demonstrates consistently superior performance in global metrics across all tested species.

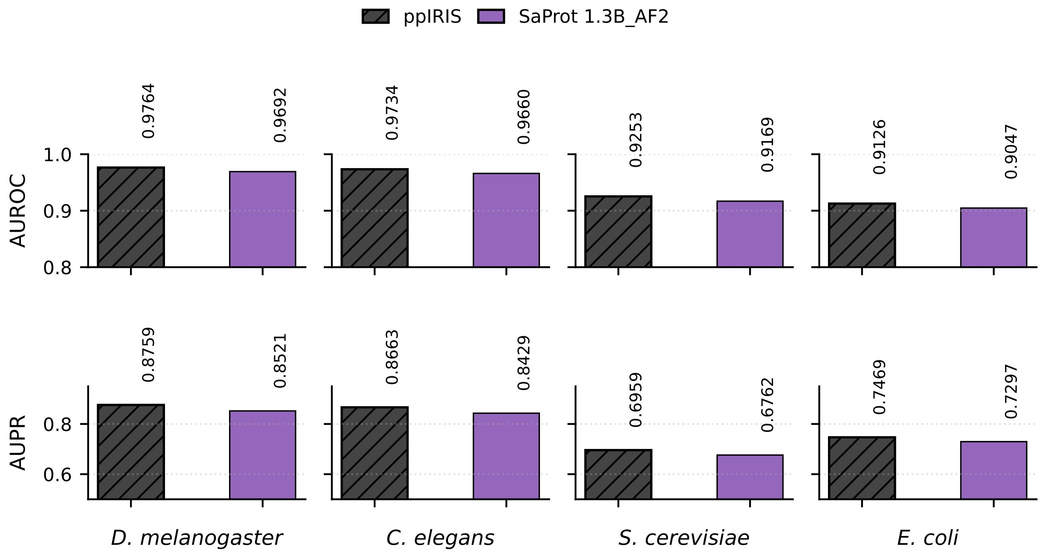

Figure S3: Performance comparison of ppIRIS and SaProt 1.3B on D-SCRIPT benchmark datasets. Bar charts display the performance of ppIRIS (black hatched bars) versus the SaProt 1.3B_AF2 baseline (purple bars) across four species: *D. melanogaster*, *C. elegans*, *S. cerevisiae*, and *E. coli*. The models are evaluated using AUROC (top row) and AUPR (bottom row). Despite SaProt being a dedicated structure-aware model, ppIRIS consistently demonstrates superior discriminative power across all tested species without requiring external structural inputs.

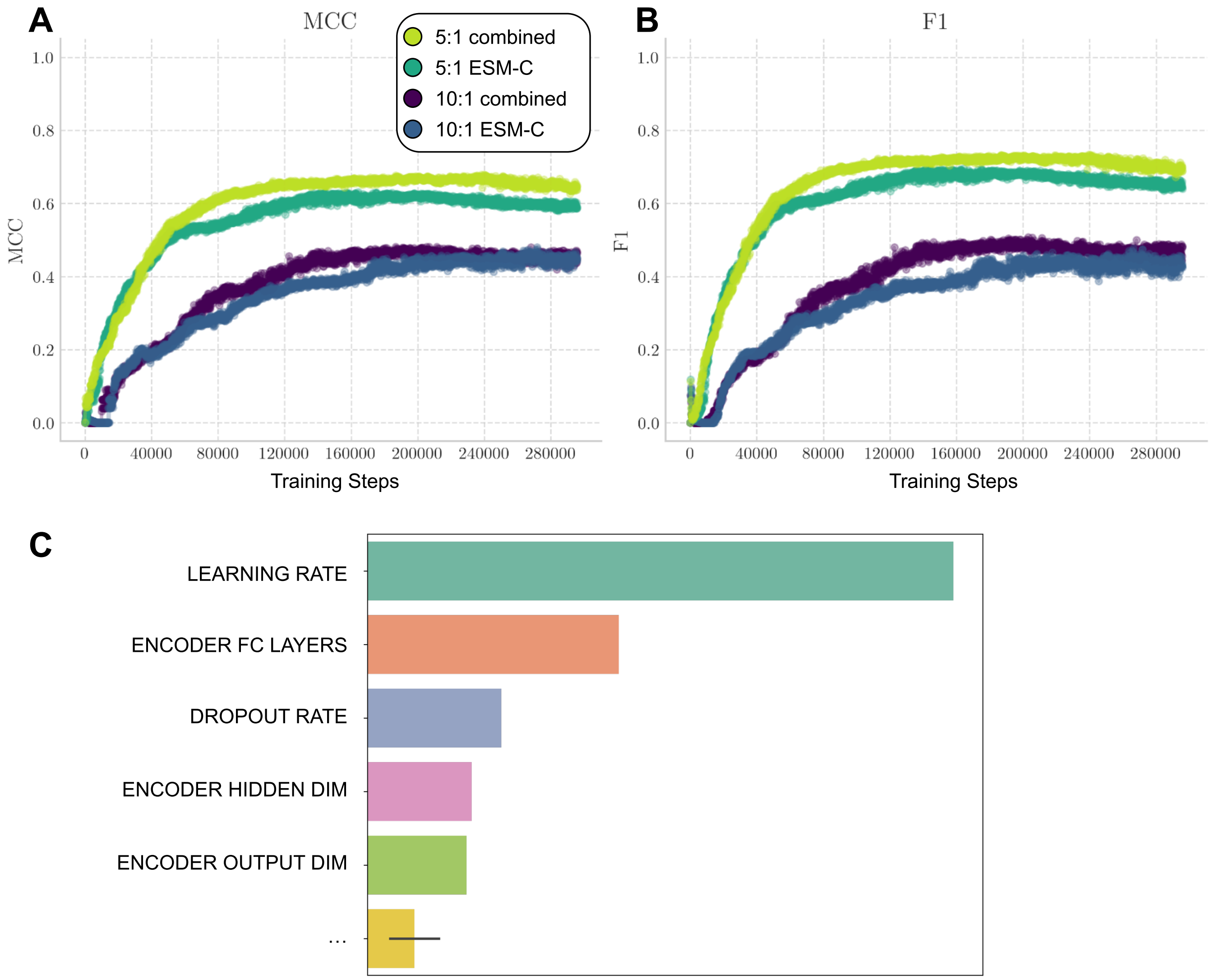

Figure S4: Prediction results for the top 10,000 intra-proteomic interactions based on the predicted PPI score. **A-B)** Validation MCC and F1 score per training step. Values in blue and purple represent the metrics for the 0:1 split using only ESM-C 300M embeddings and 10:1 split using the combined embeddings, respectively. Values in dark green are for the 5:1 ESM-only dataset, whereas light green are for the 5:1 using the combined embeddings. It becomes evident that the model is able to achieve a high F1 score and MCC, with the combined embeddings achieving the best performance. **C)** Hyperparameter importance on the classification performance, wherein the most important hyperparameters are the learning rate and the number of hidden layers in the encoder, followed by the dropout rate.

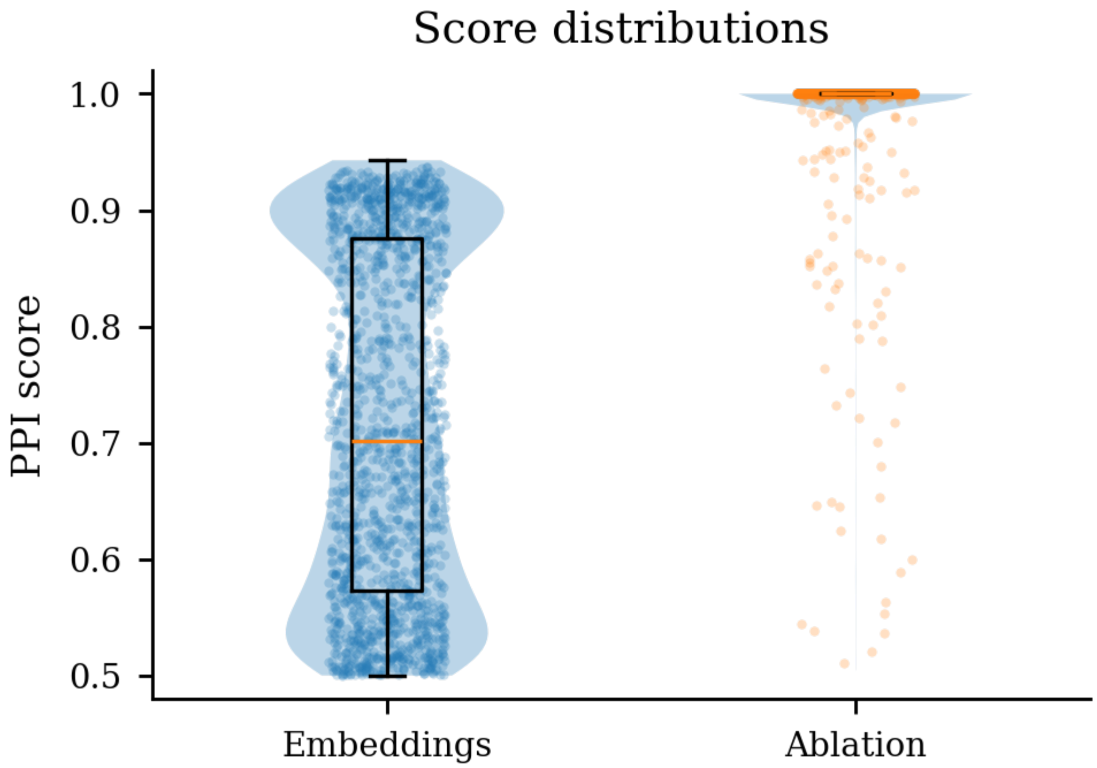

Figure S5: Score distributions using true embeddings versus random noise, for predictions with a PPI interaction score over 0.5. The model with true embeddings (blue) shows a clear distribution of predictions across several values, while the model with random noise (orange) simply predicted everything with a high score, indicating no meaningful discrimination.

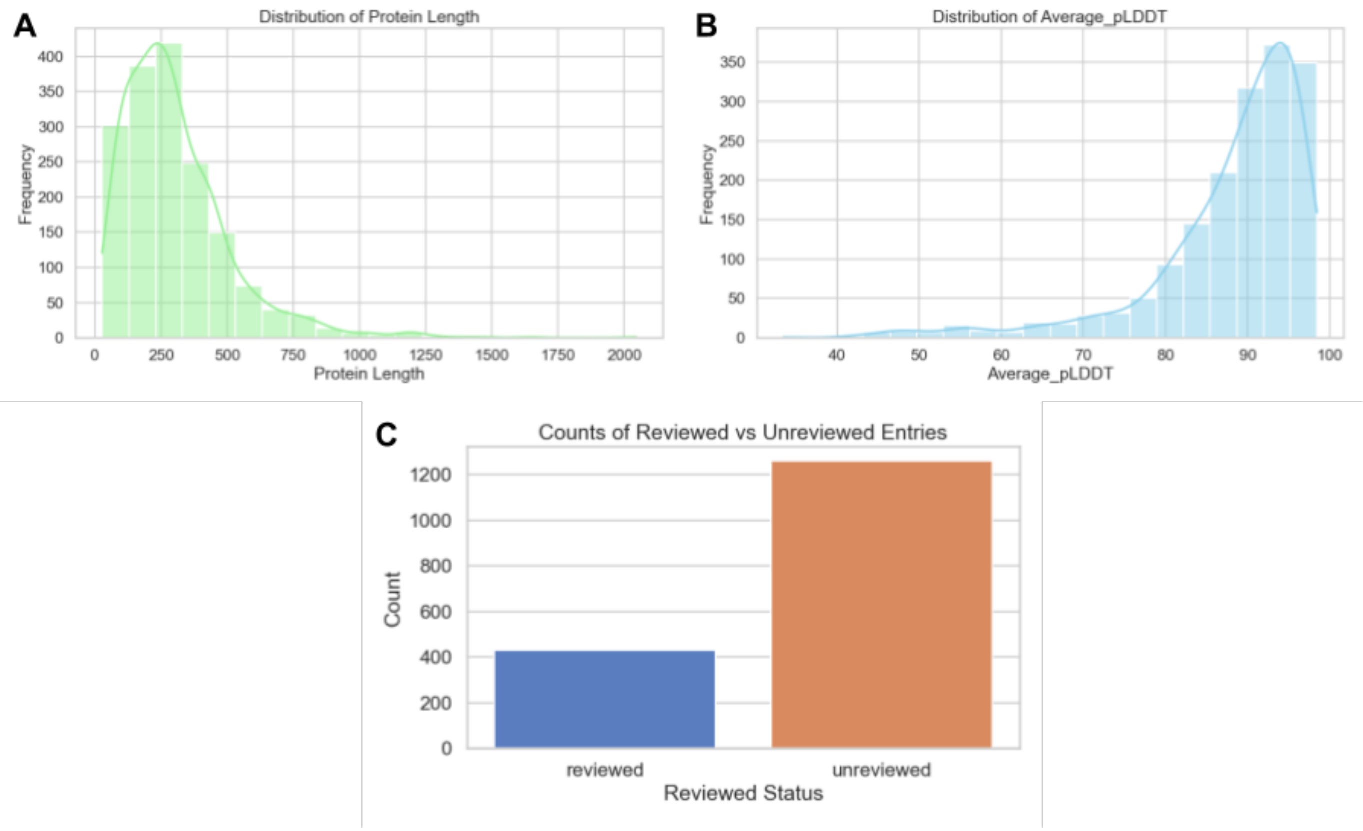

Figure S6: Characteristics of the GAS M1 Serotype proteome used for ppIRIS predictions. A) Protein length distribution showing most proteins between 100-500 amino acids. B) Distribution of average pLDDT scores across the proteome, reflecting confidence in structural predictions. C) Proportion of reviewed (manually curated) and unreviewed proteins in the proteome.

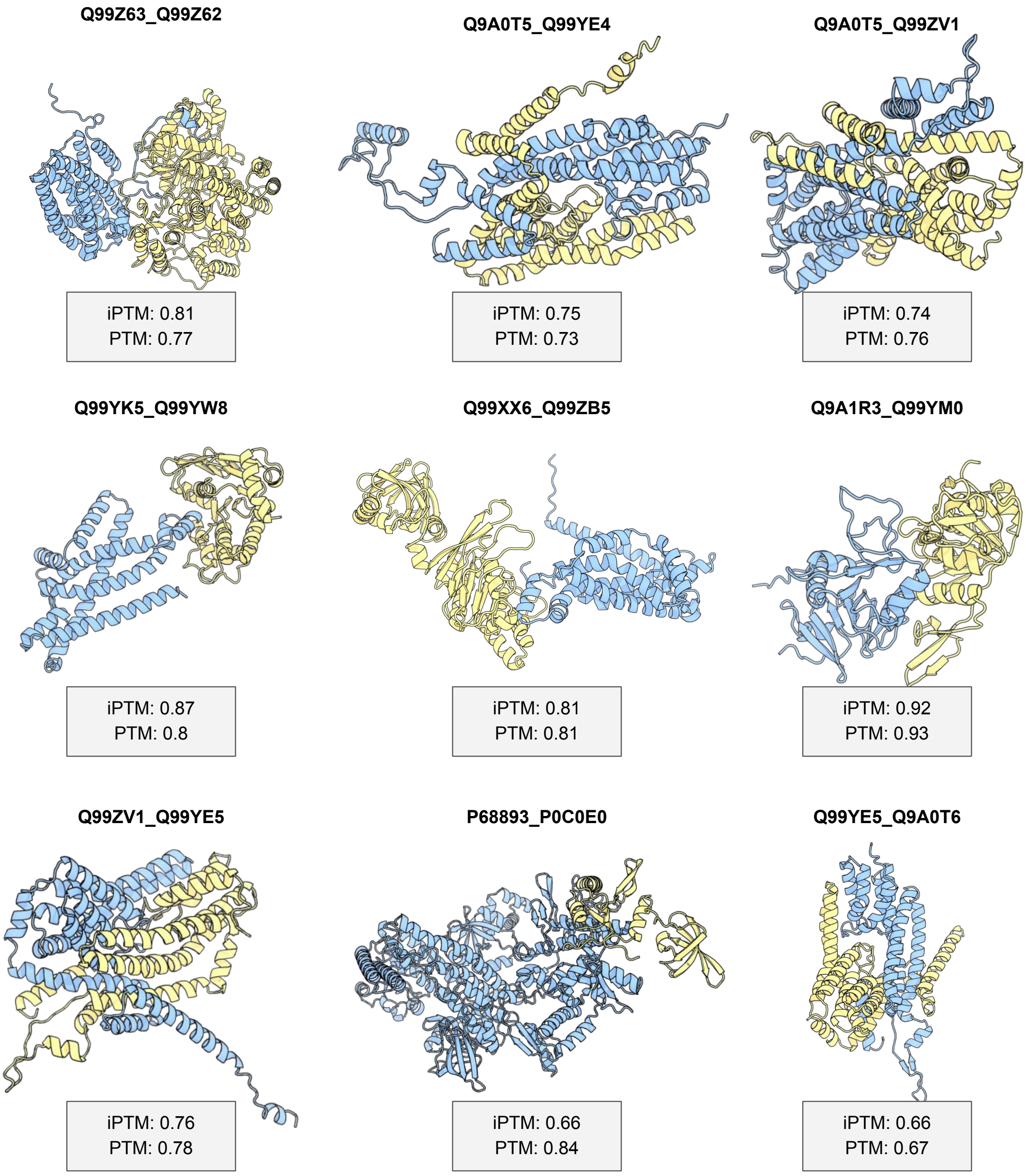

Figure S7: AlphaFold 3 models of protein-protein complexes with high confidence scores for intraproteomic interactions predictions in *S. pyogenes*.

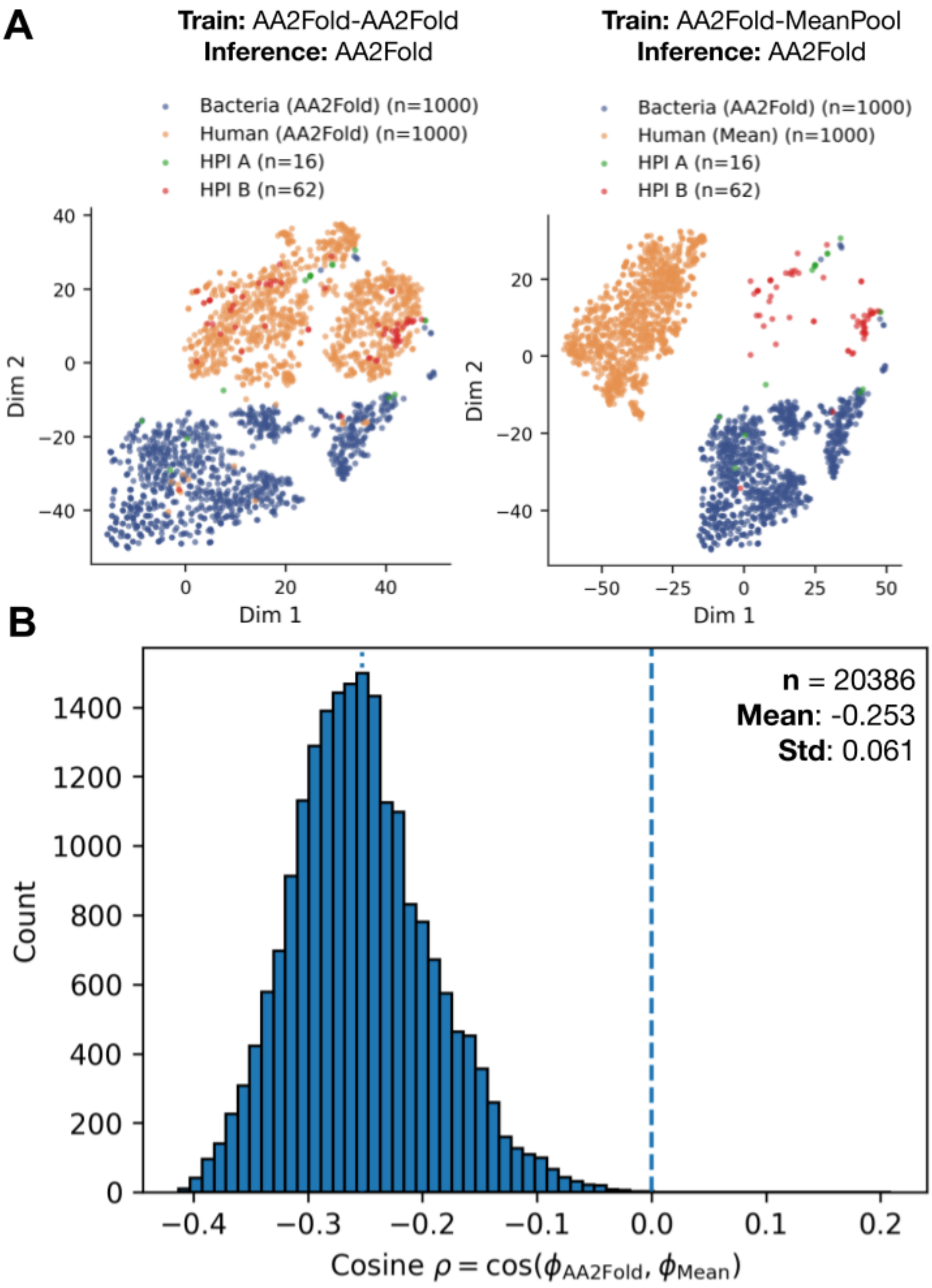

Figure S8: Intentional domain shift in cross-species predictions using different pooling strategies for ProstT5 embeddings. A) Two-dimensional tSNE projection of protein embeddings. On the left, both bacterial and human embeddings with AA2Fold-based pooling. On the right, bacterial embeddings with AA2Fold-based pooling and human embeddings with a mean pooling strategy. In both cases, the host-pathogen protein sequence embeddings were pooled with the AA2fold approach, showing a better separation from the rest when human and bacterial proteins from the training set are represented with different embeddings. B) Cosine similarity histogram between AA2Fold and MeanPool embeddings for matched proteins is broadly negative, supporting a notable difference between per-sequence embeddings obtained through different pooling approaches.

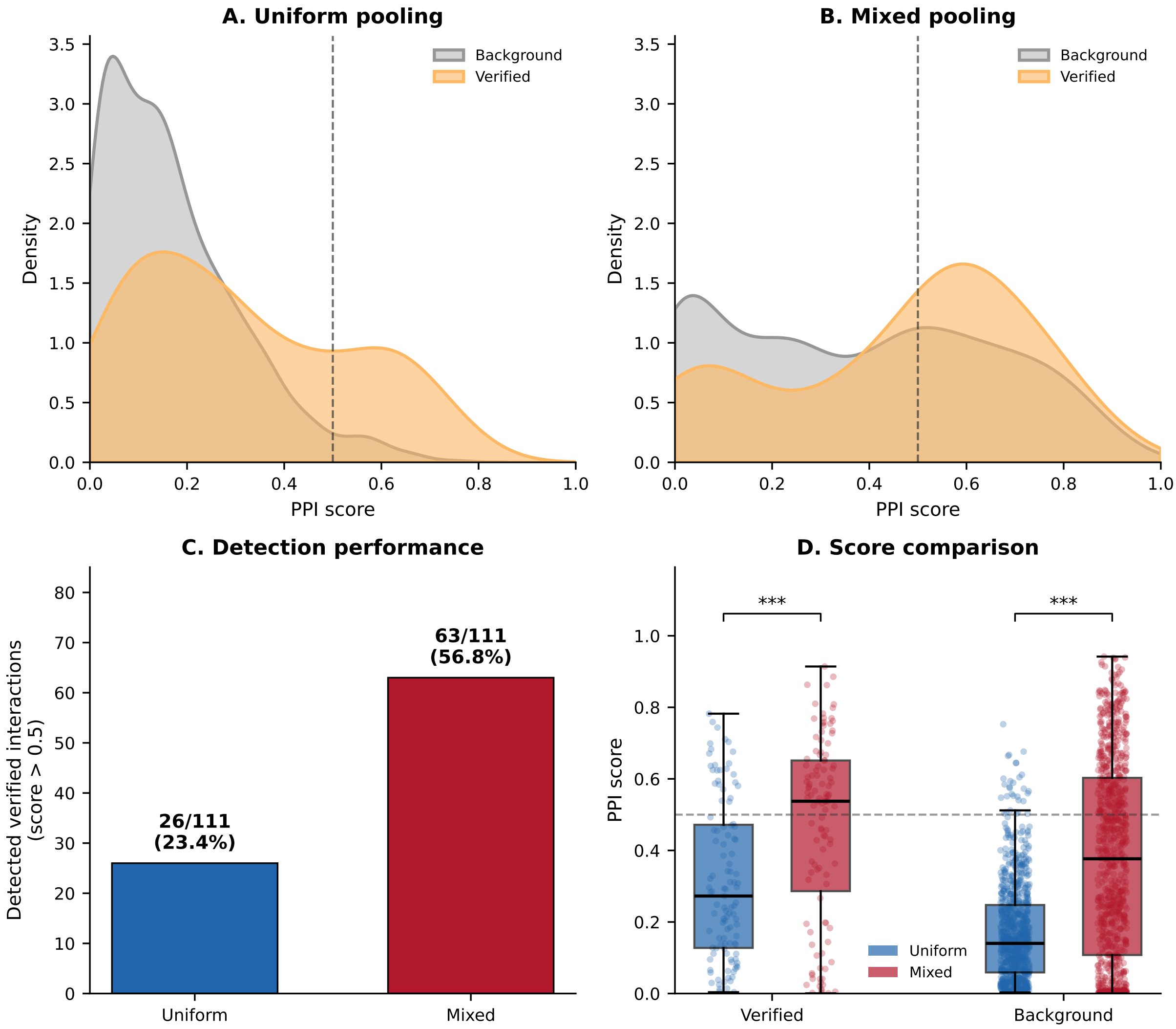

Figure S9: Ablation study of pooling strategies on host-pathogen interaction recovery. Comparison of the proposed "Mixed" pooling strategy (domain shift) versus a "Uniform" pooling baseline. **(A-B)** Density plots of prediction scores for experimentally verified interactions (orange) versus background pairs (gray). The Uniform model (A) fails to separate populations, while the Mixed model (B) successfully shifts verified interactions toward higher probabilities. **(C)** Detection performance at a score threshold of 0.5. The Mixed model recovers 63/111 (56.8%) of verified interactions, a ∼2.4-fold improvement over the Uniform model (26/111, 23.4%). **(D)** Boxplot comparison of prediction scores shows a statistically significant upranking of verified interactions using the Mixed strategy (*** $p < 0.001$, Mann-Whitney U test).

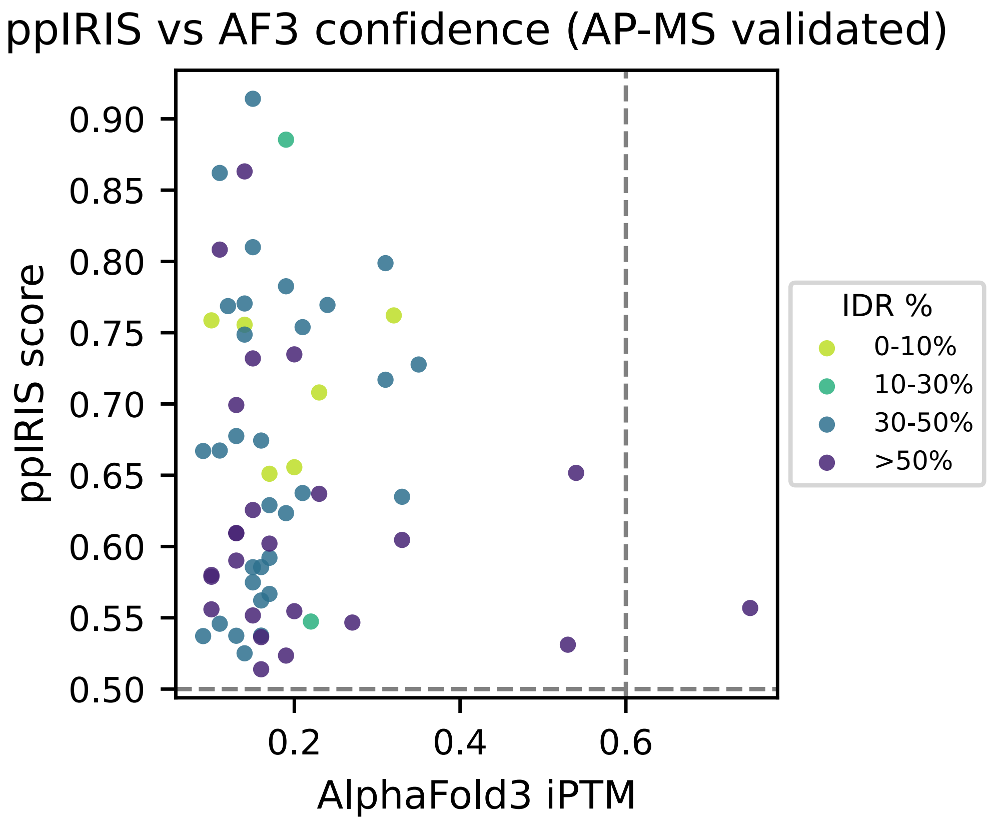

Figure S10: Comparison of iPTM and ppIRIS scores for the 63 correctly predicted interactions from **Figure 4** in the main manuscript. Each point represents a predicted interaction, with the x-axis showing the iPTM score and the y-axis showing the ppIRIS score. Points are colored by their percentage of disorder, with high disorder proteins shown in purple. The vertical dashed line indicates the AlphaFold3 iPTM threshold of 0.60.

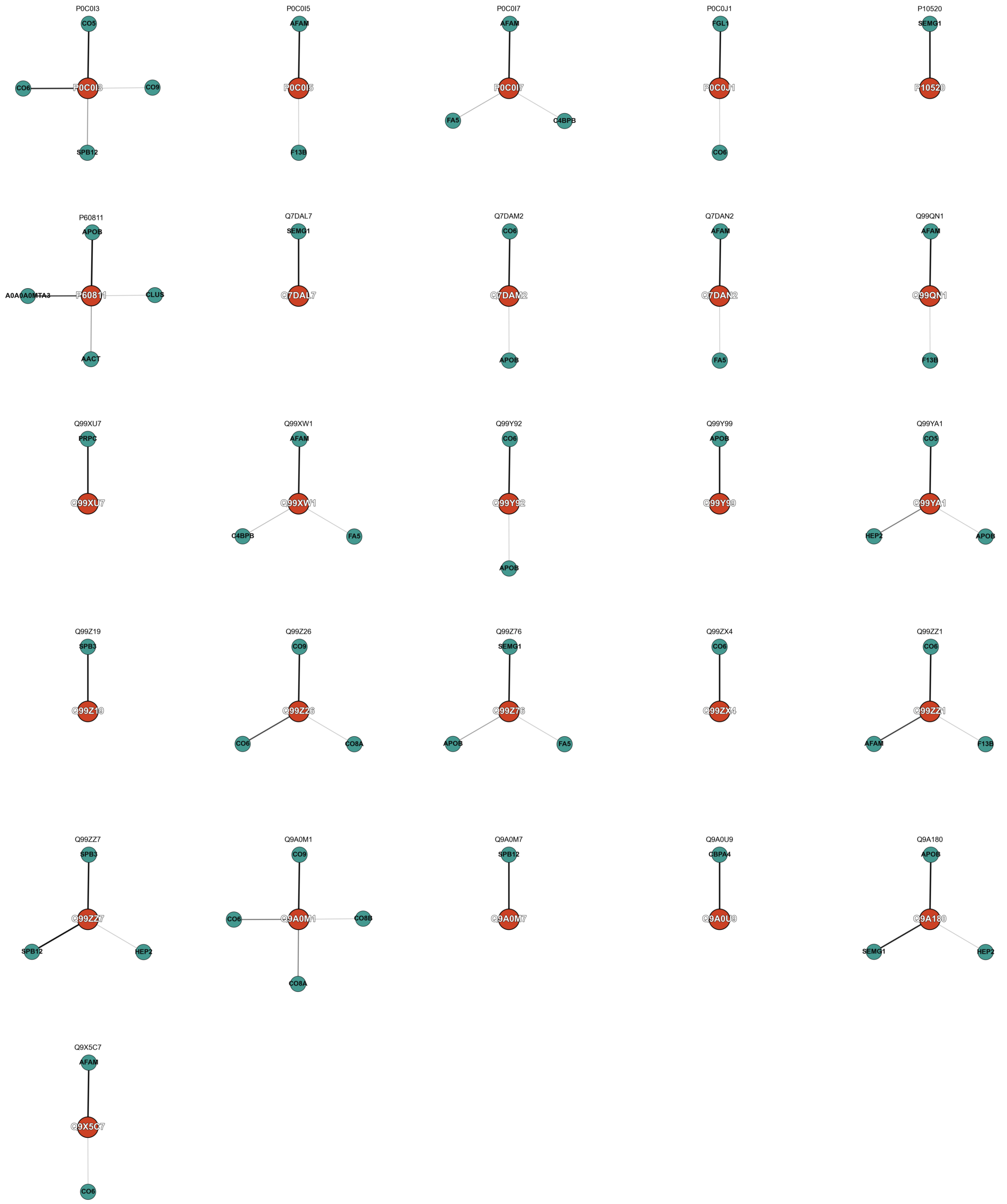

Figure S11: Subnetworks of GAS proteins with predicted interactions with human plasma proteins. The title of each subnetwork as well as the text in the central node are the UniProt IDs for that GAS protein. *S. pyogenes M1* proteins are colored red, whereas human plasma proteins are colored teal. Edges are colored by the predicted interaction score, with darker edges indicating higher confidence.

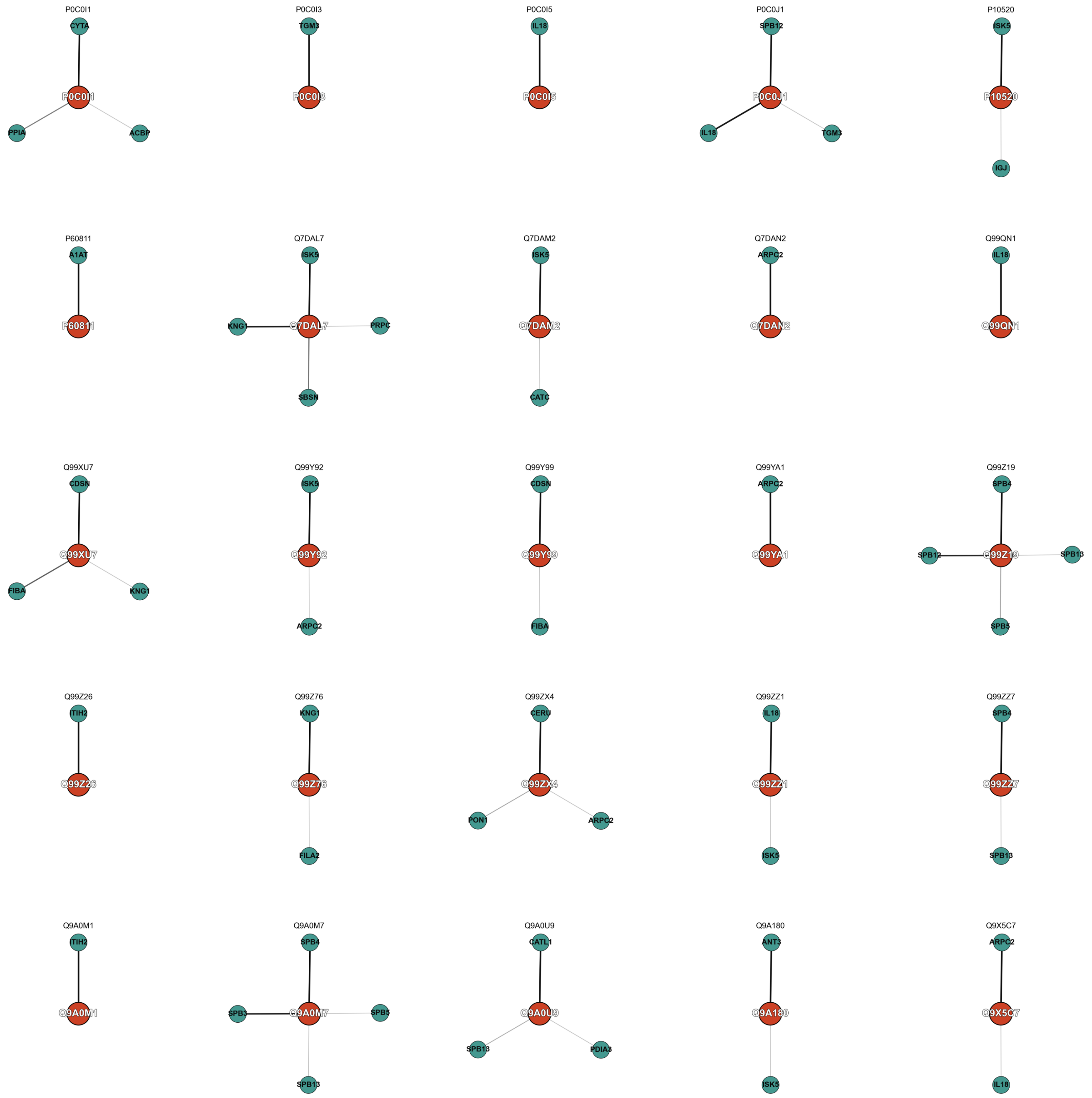

Figure S12: Subnetworks of GAS proteins with predicted interactions with human saliva proteins. The title of each subnetwork as well as the text in the central node are the UniProt IDs for that GAS protein. *S. pyogenes M1* proteins are colored red, whereas human saliva proteins are colored teal. Edges are colored by the predicted interaction score, with darker edges indicating higher confidence.

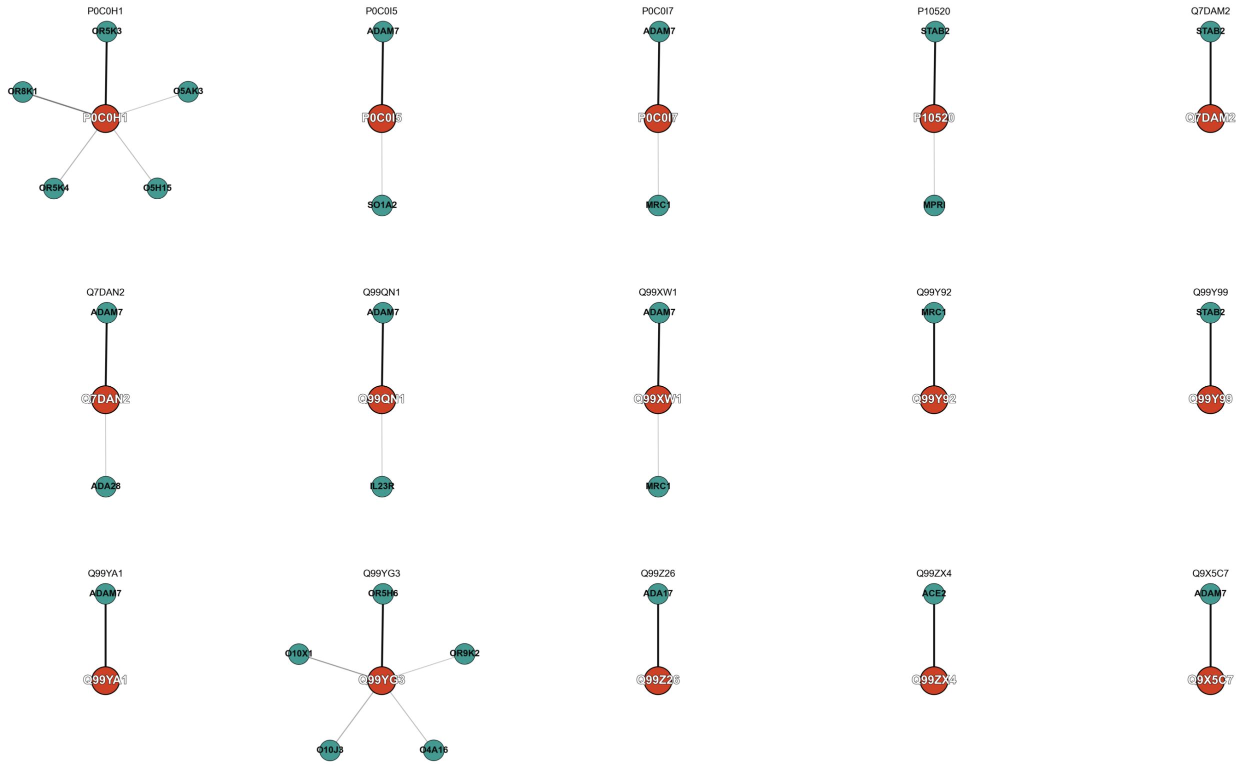

Figure S13: Subnetworks of GAS proteins with predicted interactions with human surfaceome proteins. The title of each subnetwork as well as the text in the central node are the UniProt IDs for that GAS protein. *S. pyogenes M1* proteins are colored red, whereas human surfaceome proteins are colored teal. Edges are colored by the predicted interaction score, with darker edges indicating higher confidence.

Supplementary Tables

Table S1: Cross-species PPI performance (AUROC, AUPR) comparing ppIRIS with prior sequence-based models (including Topsy-Turvy). ppIRIS uses a 0.5 decision threshold. Human results are 5-fold CV means on the D-SCRIPT dataset. The performance metrics for Topsy-Turvy, PIPR, D-SCRIPT and TUnA for all species are reported in the Topsy Turvy and TUnA manuscripts.

**Species**	**Model**	**AUROC**	**AUPR**
D. melanogaster	PIPR	0.728	0.278
	D-SCRIPT	0.890	0.605
	TUnA	0.971	0.855
	Topsy-Turvy	0.921	0.713
	**ppIRIS**	**0.976**	**0.876**
C. elegans	PIPR	0.757	0.346
	D-SCRIPT	0.853	0.550
	TUnA	0.960	0.834
	Topsy-Turvy	0.906	0.700
	**ppIRIS**	**0.973**	**0.866**
S. cerevisiae	PIPR	0.718	0.230
	D-SCRIPT	0.790	0.399
	TUnA	0.898	0.641
	Topsy-Turvy	0.850	0.534
	**ppIRIS**	**0.925**	**0.696**
E. coli	PIPR	0.675	0.271
	D-SCRIPT	0.770	0.513
	TUnA	0.883	0.677
	Topsy-Turvy	0.805	0.556
	**ppIRIS**	**0.912**	**0.747**

Table S2: Comparison of General Model Performance Metrics for Different Negative-to-Positive Ratios and Embedding Groups. All datasets were clustered at 40% sequence identity threshold.

**Embeddings**	**Split**	**W. ROC AUC**	**Avg. Prec.**	**Prec.**	**Recall**	**F1**	**MCC**
ESM-C	**5:1**	0.9401	0.9226	**0.7135**	0.6193	0.6631	0.6035
	**10:1**	0.9389	0.9216	0.6575	0.3329	0.6242	0.4420
ProstT5 3Di	**5:1**	0.8436	0.8141	0.6083	0.2847	0.3879	0.3447
	**10:1**	0.8204	0.7834	0.4217	0.0982	0.1593	0.1693
Combined	**5:1**	**0.9419**	**0.9248**	0.6998	**0.6807**	**0.6901**	**0.6292**
	**10:1**	0.9392	0.9235	0.6641	0.4203	0.5148	0.4926

Table S3: Stratified performance evaluation on conserved versus species-specific interactions. To quantify the impact of the training strategy on lineage-specific signals, a held-out bacterial dataset was partitioned based on Clusters of Orthologous Groups (COGs) assignments from STRING. Conserved interactions denote pairs where both proteins map to canonical COGs, representing widely shared bacterial machinery. Species-specific interactions involve at least one protein lacking a COG assignment, representing lineage-specific or orphan proteins. ppIRIS maintains high discriminative power across both subsets, indicating that the inclusion of diverse species in the training data does not dilute the signal for specialized, less-conserved interactions.

Subset	Count (N)	AUROC	AUPR
Conserved	1,106	0.949	0.498
Species-Specific	82	0.954	0.642

Table S4: Molecular function Gene Ontology (GO) term distribution among top 3,000 high-confidence ppIRIS predictions within the GAS M1 proteome after ribosomal proteins were removed. The table lists terms (abbreviated IDs), their definitions, and counts of associated proteins.

GO term	Definition	Count
GO:0005488	The selective, non-covalent, often stoichiometric, interaction of a molecule with one or more specific sites on another molecule.	418
GO:0003824	Catalysis of a biochemical reaction at physiological temperatures. In biologically catalyzed reactions, the reactants are known as substrates, and the catalysts are naturally occurring macromolecular substances known as enzymes. Enzymes possess specific binding sites for substrates, and are usually composed wholly or largely of protein, but RNA that has catalytic activity (ribozyme) is often also regarded as enzymatic.	225
GO:0022857	Enables the transfer of a substance, usually a specific substance or a group of related substances, from one side of a membrane to the other.	149
GO:0016787	Catalysis of the hydrolysis of various bonds, e.g. C-O, C-N, C-C, phosphoric anhydride bonds, etc.	45
GO:0140640	Catalytic activity that acts to modify a nucleic acid.	18
GO:0016740	Catalysis of the transfer of a group, e.g. a methyl group, glycosyl group, acyl group, phosphorus-containing, or other groups, from one compound (generally regarded as the donor) to another compound (generally regarded as the acceptor). Transferase is the systematic name for any enzyme of EC class 2.	10
GO:0005515	Binding to a protein.	9
GO:0016491	Catalysis of an oxidation-reduction (redox) reaction, a reversible chemical reaction in which the oxidation state of an atom or atoms within a molecule is altered. One substrate acts as a hydrogen or electron donor and becomes oxidized, while the other acts as hydrogen or electron acceptor and becomes reduced.	9
GO:0042802	Binding to an identical protein or proteins.	6
GO:0016772	Catalysis of the transfer of a phosphorus-containing group from one compound (donor) to another (acceptor).	5
GO:0140096	Catalytic activity that acts to modify a protein.	4
GO:0016746	Catalysis of the transfer of an acyl group from one compound (donor) to another (acceptor).	4
GO:0016875	Catalysis of the joining of two molecules via a carbon-oxygen bond, with the concomitant hydrolysis of the diphosphate bond in ATP or a similar triphosphate.	4

textbfGO term	**Definition**	**Count**
GO:0016462	Catalysis of the hydrolysis of a pyrophosphate bond (diphosphate bond) between two phosphate groups.	4
GO:0004812	Catalysis of the formation of aminoacyl-tRNA from ATP, amino acid, and tRNA with the release of diphosphate and AMP.	3
GO:0016874	Catalysis of the joining of two molecules, or two groups within a single molecule, using the energy from the hydrolysis of ATP, a similar triphosphate, or a pH gradient.	3
GO:0016887	Catalysis of the reaction: ATP + H2O = ADP + H+ phosphate. ATP hydrolysis is used in some reactions as an energy source, for example to catalyze a reaction or drive transport against a concentration gradient.	3
GO:0003677	Any molecular function by which a gene product interacts selectively and non-covalently with DNA (deoxyribonucleic acid).	2
GO:0016788	Catalysis of the hydrolysis of any ester bond.	2
GO:0008233	Catalysis of the hydrolysis of a peptide bond. A peptide bond is a covalent bond formed when the carbon atom from the carboxyl group of one amino acid shares electrons with the nitrogen atom from the amino group of a second amino acid.	2
GO:0016798	Catalysis of the hydrolysis of any glycosyl bond.	1
GO:0016817	Catalysis of the hydrolysis of any acid anhydride.	1
GO:0016757	Catalysis of the transfer of a glycosyl group from one compound (donor) to another (acceptor).	1
GO:0016747	Catalysis of the transfer of an acyl group, other than amino-acyl, from one compound (donor) to another (acceptor).	1
GO:0017111	Catalysis of the reaction: a ribonucleoside triphosphate + H2O = a ribonucleoside diphosphate + H+ + phosphate.	1
GO:0016684	Catalysis of an oxidation-reduction (redox) reaction in which the peroxide group acts as a hydrogen or electron acceptor.	1
GO:0042578	Catalysis of the reaction: RPO-R' + H2O = RPOOH + R'H. This reaction is the hydrolysis of any phosphoric ester bond, any ester formed from orthophosphoric acid, O=P(OH)3.	1
GO:0046790	Binding to a virion, either by binding to components of the capsid or the viral envelope.	1
GO:0008168	Catalysis of the transfer of a methyl group to an acceptor molecule.	1
GO:0140098	Catalytic activity that acts to modify RNA.	1
GO:0004518	Catalysis of the cleavage of ester linkages within nucleic acids.	1
GO:1901363	Binding to heterocyclic compound.	1

Table S5: Interaction scores (ipTM, pTM) for selected protein pairs with high MiST score from AP-MS. Protein 1 is listed with its Uniprot ID, followed by the interacting Protein 2 Uniprot ID and associated confidence metrics.

**Protein 1**	**Protein 2**	**ipTM**	**pTM**
PLG (P00747)	Q99XJ6	0.24	0.39
	P0C0B4	0.17	0.49
	P0C0F1	0.35	0.63
	P0C0I1	0.17	0.49
	P60080	0.19	0.56
	P60812	0.33	0.46
	P65123	0.24	0.55
	P66058	0.16	0.58
	P68886	0.21	0.61
	P68896	0.2	0.47
SLO (P0C0I3)	P06744	0.17	0.55
	Q08830	0.21	0.52
	Q99536	0.22	0.5
	P15153	0.11	0.54
	P13798	0.16	0.58
	P49411	0.13	0.43
	P49189	0.16	0.51
	P17213	0.17	0.47
	P52565	0.2	0.52
	O75223	0.16	0.55
C5AP (P58099)	P02810	0.23	0.71
	P20160	0.35	0.69
	P0DP57	0.33	0.72
	P0DTE7	0.22	0.6
	Q96QA5	0.29	0.63
	Q9C075	0.33	0.63
	P27482	0.37	0.71
	P00738	0.29	0.63
	P02814	0.27	0.74
	Q14574	0.2	0.51
